# Application of Artificial Intelligence to Improve Imaging in Ophthalmology

**DOI:** 10.18502/jovr.v18i1.12719

**Published:** 2023-02-21

**Authors:** Mark Christopher

**Affiliations:** Hamilton Glaucoma Center, Viterbi Family Department of Ophthalmology and Shiley Eye Institute, University of California San Diego, California, USA

Over the past decade, the literature has witnessed major advances in the field of artificial intelligence (AI). Many of these developments have focused on applying specific AI approaches, including deep learning and convolutional neural networks (CNNs), to image datasets which have greatly improved the accuracy of general image recognition and computer vision tasks.^[1]^ There has also been great interest and progress in adapting these methods for use on medical imaging data to detect disease, assess prognosis, and improve patient care.^[2]^ With respect to ophthalmic images specifically, AI models have been developed for diabetic retinopathy, macular degeneration, glaucoma, and even prediction of systemic health indicators.^[3]^ The past few years have even seen regulatory approval of autonomous AI-based systems to detect diabetic retinopathy in the US.^[4,5]^

In the current issue of *Journal of Ophthalmic and Vision Research*, Razaghi et al report the use of a deep learning approach to reduce errors in optical coherence tomography (OCT) retinal nerve fiber layer (RNFL) segmentation.^[6]^ It is known that using current standard device software, segmentation errors can be a common event.^[7,8]^ Errors in the resulting structural and thickness measurements may provide incorrect information on which diagnostic and treatment decisions could be based. Methods that provide accurate and robust segmentation methods are critical for ensuring that clinical decisions are based on correct information.

A number of investigators have approached OCT segmentation using deep learning techniques.^[9,10,11]^ These reports have typically used manual segmentation by experts on OCT data to train CNNs designed specifically for image segmentation. Based on their intended use, these algorithms can be trained to segment individual retinal layers, optic nerve head structures, or disease markers, or be programmed to perform simultaneous segmentation of all these parameters. These techniques exhibit variations in terms of modifications of the CNN architecture, training approaches, and pre-/post-processing procedures. Razaghi et al focused on providing accurate and reliable RNFL segmentation. To achieve this, they adopted a commonly used fully convolutional CNN approach (U-Net).^[12]^ They then applied post-processing steps to help clean up the segmentation and provide accurate estimates of mean RNFL thickness. When applied to their test set, they have reported performances comparable to previous studies in terms of DICE (a commonly used metric for image segmentation) and even exceeding prior results in terms of R ${}^{2}$ when comparing mean RNFL thickness to manual segmentation-based RNFL thickness values.

In summary, AI is already demonstrating a massive impact on ophthalmology (and medicine is general) that will only continue to grow. AI-based tools have the potential to impact and hopefully improve all aspects of patient care. It is critical, however, that clinical integration of these tools be performed responsibly. This includes emphasis on thorough evaluation of AI models on diverse datasets and mindfulness of their limitations.

##  Financial Support and Sponsorship

None.

## Conflicts of Interest

There are no conflicts of interest.
